# The factor structure and reliability of the Illness Attitude Scales in a student and a patient sample

**DOI:** 10.1186/1471-244X-6-46

**Published:** 2006-10-26

**Authors:** Alexander Crössmann, Paul Pauli

**Affiliations:** 1 Department of Psychology I, University of Wuerzburg, Marcusstr. 9-11, 97070 Wuerzburg, Germany

## Abstract

**Background:**

The Illness Attitude Scales (IAS), designed by Kellner in 1986, assesses fears, beliefs, and attitudes associated with hypochondriasis and abnormal illness behaviour. However, its factor structure is, especially for translations of the IAS, not sufficiently explored. Thus, the present Study aimed to analyse the factor structure of the IAS in a German student and a patient population using exploratory factor analysis.

**Methods:**

A mixed student (N = 296) and a mixed patient (N = 130) sample completed the IAS. The data was submitted to principal components analyses (PCA) with subsequent oblique rotations. From identified factor structures, scales were derived and submitted to reliability analyses as well as to a preliminary validity analysis.

**Results:**

The PCA revealed a four-factor solution in the student sample: (1) fear of illness and death; (2) treatment experience; (3) hypochondriacal beliefs; and (4) effect of symptoms. In the patient sample, the data was best explained by a two-factor solution: (1) health related anxiety and (2) effect of symptoms and treatment experience. All scales reached good to acceptable reliability coefficients. The scales derived from the student sample and those derived from the patient sample were able to distinguish between pain patients and a matched group of normal controls.

**Conclusion:**

Our data suggests that the IAS is in student samples best represented by a four factor-solution and in patient samples by a two-factor-solution.

## Background

Hypochondriasis as described in DSM-IV is a mental disorder characterized by excessive fear of or preoccupation with a serious illness, despite medical testing and reassurance to the contrary [1]. In primary care users its prevalence is estimated at 3% [2]. The disorder can significantly interfere with an individual's functioning and can be very costly to the health care system when it results in high levels of medical services utilization. To detect hypochondriasis in an early stage, it is very important that self-report questionnaires are developed and thoroughly evaluated.

One of the most popular self-report measures of hypochondriasis is the Illness Attitude Scales (IAS) by Kellner [3]. The questionnaire, which consists of 29 self-rated items, is designed to measure abnormal illness behaviour and hypochondriasis. Twenty-seven items are answered on a five-point scale, two items are answered differently. For 24 of the 27 items, the steps of the scale are labelled: 0 = no, 1 = rarely, 2 = sometimes, 3 = often, and 4 = most of the time. For the other three items, the steps of the scale are adjusted to the content of the questions.

According to the test author, the 27 five-point scale items are grouped into nine subscales of three items each. These subscales are (1) worry about illness, (2) concerns about pain, (3) health habits, (4) hypochondriacal beliefs, (5) thanatophobia, (6) disease phobias, (7) bodily preoccupation, (8) treatment experience, and (9) effects of symptoms [3].

The nine three-item subscales have shown good to marginal stability over modest time periods [3]. However, internal consistencies varied depending on the scale analysed [4]. The validity of the IAS scales has been demonstrated in a study by the test author [5]. Apart from the subscale "health habits," all IAS subscales were able to differentiate between individuals with hypochondriasis and matched family practice patients, non-patient employees, and non-hypochondriacal psychiatric patients. However, the study is limited because it is not based on an empirically derived factor structure. Despite the common usage of the IAS, only a few studies have investigated its factor structure. However, these studies are important because the nine dimensional structure of the IAS is questionable.

Most studies of the factor structure of the IAS analysed the original English version [4,6-12]. Two of them investigated student populations and found four-factor solutions. Ferguson and Daniel [4] used principal components analyses with orthogonal rotation to analyse a sample of undergraduate students (n = 101). They also used a parallel analysis [6] where the empirically derived eigenvalues were compared with randomly generated ones. This method suggested a four-factor solution: (1) general hypochondriacal fears and beliefs, (2) symptom experience and frequency of treatment, (3) thanatophobia, and (4) fear of coronary heart disease and associated health habits. In the second study, Stewart and Watt [7] used principal components analysis and oblique rotation in a sample of undergraduate students (n = 197). They also determined the number of factors by parallel analyses comparing the empirical eigenvalues with means [6] and in addition with 95th percentiles [8] of the random data. This procedure led to the following factors: (1) general fear of illness, death, disease, and pain; (2) behaviour motivated by illness concerns; (3) specific hypochondriacal beliefs and disease conviction; and (4) disruptive effects of symptoms.

However, two other studies reported five-factor solutions. Hadjistavropoulos et al. [9] used principal components analysis (PCA) and confirmatory factor analysis (CFA) to investigate the factor structure of the IAS in a student sample (n = 780). Using half of the sample (n = 390) to perform the PCA, the parallel analyses [6,8] suggested a five-factor solution. The oblique rotated factors were: (1) fear of illness, death, disease and pain; (2) effects of symptoms; (3) treatment experiences; (4) disease conviction; and (5) health habits. The authors then performed a confirmatory factor analysis with the second part of the sample (n = 390). They tested the two four-factor solutions we mentioned earlier [4,7] and the factor solution from the first part of their study. The model from the first part of their study and a modification of it (factor "health habits" was deleted) fitted the data best. However, considering recommendations for the goodness-of-fit indices [10], none of the models reached the required standards.

Cox et al. [11] replicated these findings with another student sample (n = 309). They found a factor structure similar to the one in the study by Hadjistavropoulos et al. [9], and also extracted two higher order factors ("Health Anxiety" and "Health Habits"). The second study, which investigated chronic pain patients (n = 197) [12], found the following five-factor solution: (1) worry about illness and pain, (2) symptom effects, (3) health habits, (4) disease phobia and conviction, and (5) fear of death. The investigators used PCA with oblique rotation. The number of components was determined by parallel analysis.

Only three studies analysed translations of the IAS. Speckens et al. [13] administered a Dutch translation to three samples of subjects: (a) general medical outpatients (n = 130), (b) general practice patients (n = 113), and (c) the general population (n = 177). Using the Kaiser criterion and the scree test [14], they found a two-factor solution in all of the samples: (1) health anxiety and (2) illness behaviour. Dammen et al. [15] administered a Norwegian translation to a sample of cardiological out-patients (n = 199). The investigators used principal components analysis with following varimax rotation. The number of factors was determined by the Kaiser criterion and the examination of the scree plot. This procedure revealed three factors: (1) health anxiety, (2) illness behaviour, and (3) health habits. Hiller et al. [16] administered a German translation of the IAS to a mixed sample of patients with mental and psychophysiological disorders. Using a principal component analysis and subsequent varimax rotation they found a two factor solution. To determine the number of factors, the authors first calculated the Kaiser criterion, which revealed 7 factors, but then they decided for the two factor solution because of interpretability of the factors. This solution is highly similar to the one of Speckens et al. [13].

The reported studies showed convincing evidence that the structure of the IAS is less complex than the one proposed by the test author [3]; however, no common factor-solution has been found. In addition, the results of these studies differ with regard to the number of factors as well as the items assigned to the factors. Unfortunately, interpreting these differences is difficult because the studies differ in both design and methodology. The three studies, which investigated translations of the IAS, suggested less than four factors. However, they only used the Kaiser criterion and the scree-test to determine the number of factors [13,15]. The only study that analysed a German translation of the IAS used the Kaiser criterion and the interpretability of the factor solution to determine the number of factors [16]. Unfortunately, the Kaiser criterion and the scree-test are problematic [17,18]. The Kaiser criterion, which only considers factors with an eigenvalue greater than one, normally leads to an overestimation of the number of factors because it falsely assumes that a factor with an eigenvalue greater than one is substantial. On the other hand, the Kaiser criterion can also lead to an underestimation of the number of factors because factors with initial eigenvalues lower than one can gain higher eigenvalues after the rotation of the factor solution. The scree-test, which searches for demarcations in the plot of the eigenvalues, is especially problematic in plots where no demarcation exists or where more than one demarcation exists.

Thus, only a few studies have investigated the factor structure of the IAS with valid statistical methods[7,9,19] and none investigated the non-Englisch versions of the IAS. However, the factor-analytic evaluation of the none-English versions of the IAS is very important because these translations are widely used. Therefore, the present study aimed to analyse the factor structure of a German translation of the IAS [20]. It also evaluated the reliability and stability of the factor analytic based subscales and assessed the ability of these scales to discriminate between chronic pain patients and normal controls.

## Methods

The study protocol, as described in this section, complies with the Helsinki Declaration. All participants included in the analysis were informed in writing about the study and agreed to participate.

### Participants

The data of two mixed samples was used for explorative factor analyses. The first was a sample of undergraduate psychology and theology students (N = 296) of which 67.9 % were female. The average age of the sample was 24.7 years (SD = 5.3 years) years. All students were recruited in lectures. The second sample was a mixed group of psychosomatic patients, chest pain patients, chronic headache patients, and general practitioner patients who were suspected to suffer from hypochondriasis (N = 130). All patients were recruited at the health institution from which they were seeking help, and they had in common the fact that their symptoms could not be explained by any medical condition. Of these patients 49.2% were female and the average age was 45.2 years (SD = 12.5 years).

For the validity analysis, a sample of normal controls (n = 29, 48% female, average age = 38.6; SD = 13.7) was recruited that matched the chronic headache patients who were part of the mixed patient sample. Of the headache patients 44.4 % were female (n = 27) and there average age was 36.4 years (SD = 13.2 years).

### Procedure

In the student sample, the Illness Attitude Scales was administered in either group session in a lecture theatre (psychology students, n = 169) or individual testing sessions (theology students, n = 129). In the patient group and in the normal control sample, the IAS was administered in single testing sessions. In order to estimate the test-retest reliability, 112 psychology students (part of the mixed student sample) filled out the IAS three times over a four weeks period.

### Data analysis

The factor structure of the IAS was analysed by a explorative principal components analysis (PCA). We chose this procedure after a previous confirmatory factor analysis in which none of the models from the literature fitted the data adequately. The principal components analysis is a widely used and well evaluated technique to find underlying dimensions in a set of variables [21]. We chose it also for comparison reasons because it is the only factor analytic method previous studies used to evaluate the factor structure of the IAS. Oblique rotation (Oblimin, δ = 0) was used because the original IAS had been designed as dependent scales, and recent studies found a better data fit for dependent factor solutions [9].

The number of factors was determined by parallel analyses [6,8] as well as the minimum average partial method (MAP) [22]. Following recommendations made by Longman et al. [8], the eigenvalues extracted from the empirical data were compared to the means and 95th percentiles of the eigenvalues extracted from normally distributed random data. In accordance with recent suggestions by O'Connor [23], eigenvalues were also extracted from data constructed from random permutations of the empirical variables. This process produces correlations between the variables that scatter around zero; however, the distributions of the original variables are preserved.

Subscales were constructed from factor loadings (>.40 and difference to loadings on other factors >.15) and their reliability and stability were analysed. Consequently, we excluded hyperplane items and items with complex loadings. Student t-tests and discriminant analysis were performed to estimate the validity of these scales.

## Results

### Student sample

The Kaiser-Meyer-Olkin measure of sampling adequacy [24] with a value of 0.83, as well as Bartlett's test of sphericity (which was highly significant) indicated that the IAS in the student sample were appropriate for principal components analyses (PCA). Applying PCA, the eigenvalues for the first ten components were 6.40, 2.51, 1.87, 1.73, 1.23, 1.14, 1.02, 0.90, and 0.83. Cattell's scree test, which was only used as a screening method because of its lack of reliability [18], suggested a four-factor solution. The parallel analyses [6,8] using the mean and the 95th percentile indicated that the data were best explained by a four-factor solution. This was due to the extraction of the eigenvalues from the random data as well as from the permuted original data [23]. The minimal average partial method [22] also revealed a four-factor solution. Hence, the four factors were extracted and oblimin rotated (δ = 0). This model accounted for 46.3 % of the variance. The correlations between the factors ranged from 0.04 to 0.35.

Based on the factor loadings, the IAS items were assigned to scales, which had (1) 9 items, (2) 4 items, (3) 3 items, and (4) 4 items (see Table 1). The remaining six items could not be assigned to scales. Of these, two had no significant loadings on the four factors and four had significant loadings on two scales. The scales were named (1) fear of illness and death, (2) treatment experience, (3) hypochondriacal beliefs and (4) effect of symptoms and attained reliabilities [25] of (1) α = 0.82, (2) α = 0.75, (3) α = 0.65 and (4) α = 0.74 (n = 296). The inter-item correlations reached values of (1) 0.35, (2) 0.45, (3), 0.39 and (4) 0.43. The test-retest correlations in a group of psychology students (n = 112) for a three week interval were 0.87, 0.85, 0.63 and 0.72. Applying the reported scaling procedure to the data from the mixed patient sample, we found internal consistencies, (0.88, 0.64, 0.61, and 0,70) similar to the student sample. Only the scale treatment experience had a slightly lower value.

**Table 1:** Factor Loadings of IAS Items

	**Students**				**Patients**	
**Item number and description**	**F1**	**F2**	**F3**	**F4**	**F1**	**F2**

2. Are you worried that you may get a serious illness in the future?	0.76^a^	0.17	0.20	0.27	0.79^a^	0.12
3. Does the thought of a serious illness scare you?	0.72^a^	0.24	0.09	0.21	0.81^a^	0.03
4. If you have pain. do you worry about a serious illness?	0.68^a^	0.02	0.33	0.28	0.78^a^	0.02
16. Are you afraid that you may have cancer?	0.67^a^	-0.09	0.21	0.29	0.54^a^	0.27
15. Are you afraid that you may die soon?	0.63^a^	-0.02	0.09	0.24	0.61^a^	0.11
13. Are you afraid of news which reminds you of death?	0.57^a^	0.05	-0.11	0.18	0.60^a^	0.02
1. Do you worry about your health?	0.56^a^	0.32	0.37	0.23	0.71^a^	0.21
14. Does the thought of death scare you?	0.56^a^	0.11	-0.17	0.15	0.57^a^	-0.01
6. If a pain lasts a week or more, do you believe you have a serious illness?	0.51^a^	0.04	0.32	0.25	0.75^a^	0.17
23. How often do you see a doctor?	0.30	0.71^a^	0.06	0.34	0.41	0.54
24. How many different caregivers have you seen in the past year?	0.23	0.69^a^	0.15	0.30	0.05	0.63^a^
25. How often have you been treated in the last year?	0.23	0.67^a^	0.18	0.38	0.13	0.59^a^
5. If a pain lasts for a week or more do you see a physician?	0.14	0.61^a^	0.06	0.17	0.34	0.27
10. Do you... that you have a disease... but doctors have not diagnosed it?	0.28	-0.05	0.64^a^	0.37	0.63^a^	0.08
12. When your doctor tells you what was found, do you believe you have it?	0.34	-0.08	0.63^a^	0.23	0.56^a^	0.19
11. When your doctor says you have no disease, do you refuse to believe it?	0.13	0.06	0.47^a^	0.31	0.36	-0.02
28. Do your bodily symptoms stop you from concentrating?	0.20	0.23	0.15	0.84^a^	0.19	0.70^a^
27. Do your bodily symptoms stop you from working?	0.07	0.21	0.12	0.77^a^	0.08	0.61^a^
29. Do your bodily symptoms stop you from enjoying yourself?	0.29	0.03	0.17	0.74^a^	0.45	0.68^a^
20. When you notice a Sensation.... do you find it difficult to think of...?	0.29	0.16	0.12	0.50^a^	0.56^a^	0.11
7. Do you avoid habits which may be harmful to you, such as smoking?	-0.19	0.42	0.33	-0.26	-0.01	-0.16
8. Do you avoid foods which may not be healthy?	-0.21	0.28	0.52^b^	-0.16	0.07	-0.34
9. Do you examine your body to find whether there is something wrong?	0.14	0.41	0.55	0.16	0.52^a^	-0.12
17. Are you afraid that you may have heart disease?	0.33	-0.24	0.21	0.22	0.31	-0.42
18. Are you afraid that you may have another serious illness?	0.57	-0.06	0.52	0.26	0.59^a^	0.29
19. When you read or hear about an illness, do you get similar symptoms?	0.39	-0.13	0.39	0.29	0.49^a^	0.03
21. When you feel a sensation in your body, do you worry about it?	0.45	0.23	0.35	0.52	0.75^a^	-0.07

Factor 1: fear of illness and death; Factor 2: treatment experience; Factor 3: hypochondriacal beliefs; Factor 4: effect of symptoms

^a ^Loadings that can be explicitly assigned to a single factor (>.40 and difference to loadings on other factors >.15)

^b ^Despite its loading on the factor "hypochondriacal beliefs," item 8 is not assigned to it because of its negative impact on the reliability of the scale.

In all the scales, the means were significantly greater (p < 0.0001) in chronic headache patients than in normal controls (see Table 2). To further examine the validity of the scales, discriminant analysis has been applied to separate the group of chronic headache patients from the matched normal controls. Using the four factor analytic scales as predictors, only one subject of each group was falsely assigned (96.4% correctly classified). To prevent overestimation, the jackknife classification [18] was used. In this process, the data from the classified case is left out and then is repeated for each case. The loadings of the discriminant function (the correlations between the predictors and the function) show that the "effect of symptoms" scale (0.88) had the most influence on the function, followed by the "treatment experience" scales (0.48). These two scales enabled the correct classification of 96.4% of the subjects.

**Table 2:** Scale means of chronic pain patients and normal controls

		Chronic headache patients		Normal controls	
		
Scale	Maximum possible score	M	SD	M	SD
Fear of illness and death	36	12.11	5.80	5.07	4.11
Treatment experience	16	11.63	2.44	5.45	3.00
Hypochondriacal beliefs	12	2.26	1.83	0.24	0.58
Effect of symptoms	16	9.37	2.48	1.03	1.43

Note. Differences between factor scores are significant on 0.0001 levels (2-tailed) for all four factors.

### Patient sample

In the mixed patient sample (N = 130), the KMO reached a value of 0.81, and therefore, the sample could be regarded as suitable for PCA. The data set's first ten eigenvalues were 7.64, 2.70, 1.85, 1.50, 1.33, 1.22, 1.15, 0.97, 0.94, and 0.88. The parallel analysis suggested a three-factor solution, whereas the minimal average partial method suggested only two factors. However, the two-factor solution, which accounted for 38.3% of the variance, was easier to interpret. Therefore, we extracted two factors, which we subsequently subjected to oblimin rotation.

Based on the factor loadings we assigned the IAS items to two scales, which comprised (1) 16 items and (2) 5 items. Of the remaining items, four items had no loadings greater than 0.40 while two items loaded significantly on both scales (see table 1). The two scales were named (1) "health related anxiety" and (2) "effect of symptoms and treatment experience." In the patient sample, internal consistencies of the two scales reached values of (1) α = 0.90 and (2) α = 0.73.

A comparison of the factor solution derived from both the student and the patient sample shows that the first factor (health related anxiety) from the patient sample completely includes the first factor of the student sample. However, the first factor from the patient sample also comprises items from the original Kellner scales "bodily preoccupation" and „hypochondriacal beliefs" as well as two other items (Nos. 9 & 18). Like two items from the Kellner scale "bodily preoccupation" (Nos. 18 & 19) these two items were, unassigned in the student sample factor solution. The second factor (effect of symptoms and treatment experience) include the Kellner scale "effect of symptoms" and two items of the scale "treatment experiences".

## Discussion

The results of the present study suggest that the structure of the IAS in undergraduate students is best explained by a dependent four-factor solution, which factors can be named (1) "fear of illness and death," (2) "treatment experience", (3) "hypochondriacal beliefs," and (4) "effect of symptoms". Consistent with other factor analytic investigations in different samples [4,7,9,13,15,19], a factor solution less complex than the one proposed by Kellner [3] was found. The number of important factors, in the student sample found is consistent with two previous studies [4,7]; however, even these studies differ according to item-factor assignments.

The factor solution of the present student sample most resembled a revised four-factor solution by Hadjistavropoulos et al. [9], where the factor "health habits was omitted". In both studies, the scales "treatment experience" and "effect of symptoms" were comprised of the same items. Both scales consisted of the eponymous original IAS [3] plus one additional item. The "fear of illness and death" scale of the present study and the "fear of illness, death, disease and pain" scale from Hadjistavropoulos et al. [9] have seven items in common. These include the original "thanatophobia" items, two items of the original "worry about illness" scale (which is complete in the present study) [3], as well as two other items. The "hypochondriacal beliefs" scale of the present study is identical to the eponymous Kellner scale [3]. The corresponding scale in the revised solution of Hadjistavropoulos et al. [9] is very similar, but it contains one additional item.

The four-factor solution derived in this study explained 46.3 % of the variance of the sample. This is equal to the variance explained by the four-factor solution of Dammen et al. [15]; however, it is less than that found by Stewart and Watt [7] in their four-factor solution (53.9%). The five-factor solutions [9,19] also accounted for more variance (52 % and 53 %).

The alpha coefficients [25] of the scales, which ranged from 0.82 to 0.65 are comparable with those achieved in other studies [9]. The 9-item scale, "fear of illness and death," achieved the greatest value. The 4-item scales ("treatment experience" and "effect of symptoms") reached values slightly lower than that of the first scale. The least internal consistency was found for the "hypochondriacal beliefs" scale comprising only three items. The inter-item correlations (0.35, 0.45, 0.39, and 0.43) revealed that the alpha coefficients were mostly influenced by the scale length. Thus, more items should be added to the last three scales in order to improve their reliability. Comparing the alpha coefficients (0.87 to 0.63) and test-retest correlations, the four scales appeared to be stable over a period of three weeks. The lack of correlation can be traced to the lack of internal consistency.

The scales derived from the student sample also reached acceptable internal consistencies in the patient's sample. However, the principal component analysis of the patient sample suggests a simpler structure than the one of the student sample. The first factor (health related anxiety) included the items of the first scale from the student sample completely but also comprised items from other scales. The second factor (effect of symptoms and treatment experience) included two of the original Kellner scales. The results of the present study would therefore suggest a simpler factor structure in patients than in students. Previous studies did not indicate that the IAS forms a simpler structure in patients than in students or normal controls. However, this might be due to the lack of studies that investigated both groups with the same methods.

Two other studies reported a two-factor solution [13,16] in translation of the IAS. Speckens et al. [13] investigated three different samples, including patients and normal controls. Hiller et al. [16] investigated a mixed German patient sample. Another study found a three-factor solution in a translation of the IAS administered to cardiology out-patients [15]. However, all of the studies used only the Kaiser criterion and the scree test to determine the number of factors. In addition, two studies using the Kaiser criterion suggested six [15] and seven [16] factors, but the researchers decided for a three- and a two-factor solution respectively. Those results are therefore difficult to compare with our results.

The significant differences in scale means between chronic headache patients and normal controls were consistent with findings of Kellner et al. [5], where eight of the nine original scales [3] showed significant differences between hypochondriacal patients and other non-hypochondriacal samples. In addition, the present study could demonstrate that a discriminant function, based on the scales derived from the four-factor solution, could adequately classify chronic headache patients and normal controls. The scales "effect of symptoms" and "treatment experience" had the most impact on the function. Although this finding underpins the validity of the IAS, further research is needed in this field. Comparisons are needed, not only between patients and controls, but also between groups of patients, with or without organic causes for their complaints. In addition, the ability to predict the outcome of medical investigations should be examined.

A limitation of the present study is the relatively small sample size of the mixed patient sample. However, the number of subjects was almost five times the number of items and the Kaiser-Meyer-Olkin indicated that the data was suitable for this PCA. A limitation of the suggested scoring procedure is the fact that a significant number of items (n = 6) cannot be incorporated because (1) they had no significant loadings (hyperplane), or (2) they had two significant loadings (complex structure). This violation of Thurston's demand for simple structure is equally strong in the four-factor student as in the two-factor patient solution.

Further empirical studies are needed to explore how the number of factors is influenced by the nature of the sample (e. g. cardiology versus headache versus general population). Emphasis should be put on comparable methodology like factor analytic methods for extraction, number of factors, and rotation. This research could lead either to a single factor solution or to different scoring rules for different groups. Once a satisfying structure has been found, research should put emphasis on the differential validity of the IAS subscales.

Although the original structure could not be confirmed, some of the problems in finding a simpler common factor structure might arise from the fact that the IAS was not constructed to form a structure simpler than the original nine scales. Therefore, researchers should also consider alternative ways to construct assessment instruments for hypochondriasis and illness behaviour. One attempt is to construct a questionnaire based on theoretically founded aspects of hypochondriasis and then try to confirm its structure empirically [26].

## Conclusion

Even though there is a further need to investigate the IAS and develop other instruments, we can recommend its use in applied scientific studies. The German translation can be scored according to the factor solution of the present study. In a patient sample, we suggest the two-factor solution, whereas in a student sample and in comparisons between students and patients, we suggest the four-factor solution.

## Competing interests

The author(s) declare that they have no competing interests.

## Authors' contributions

AC prepared and conducted the study as part of his dissertation. He also analysed the data and prepared the manuscript. PP supervised the preparation, conduction and evaluation of the study, and participated substantially in preparing the manuscript. All authors have read and approved the final manuscript.

## Pre-publication history

The pre-publication history for this paper can be accessed here:


